# Spermidine enhances the efficacy of adjuvant in HBV vaccination in mice

**DOI:** 10.1097/HC9.0000000000000104

**Published:** 2023-03-24

**Authors:** Daisuke Ito, Hiroyasu Ito, Tatsuya Ando, Yasuhiro Sakai, Takayasu Ideta, Ken J. Ishii, Tetsuya Ishikawa, Masahito Shimizu

**Affiliations:** 1 Department of Gastroenterology, Gifu University Graduate School of Medicine, Gifu City, Japan; 2 Department of Joint Research Laboratory of Clinical Medicine, Fujita Health University School of Medicine, Aichi, Japan; 3 Department of Gastroenterology, Central Japan International Medical Center, Gifu, Japan; 4 Division of Vaccine Science, Department of Microbiology and Immunology, The Institute of Medical Science, The University of Tokyo, Tokyo, Japan; 5 Laboratory of Adjuvant Innovation, Center for Vaccine and Adjuvant Research Center, National Institutes of Biomedical Innovation, Health and Nutrition, Osaka, Japan; 6 Department of Integrated Health Sciences, Nagoya University Graduate School of Medicine, Nagoya, Japan

## Abstract

**Background:** Various vaccine adjuvants have been developed to eliminate HBV from patients with chronic HBV infection. In addition, spermidine (SPD), a type of polyamine, has been reported to enhance the activity of immune cells. In the present study, we investigated whether the combination of SPD and vaccine adjuvant enhances the HBV antigen-specific immune response to HBV vaccination. **Methods:** Wild-type and HBV-transgenic (HBV-Tg) mice were vaccinated 2 or 3 times. SPD was orally administered in drinking water. Cyclic guanosine monophosphate–AMP (cGAMP) and nanoparticulate CpG-ODN (K3-SPG) were used as the HBV vaccine adjuvants. The HBV antigen-specific immune response was evaluated by measuring the HBsAb titer in blood collected over time and the number of interferon-γ producing cells by enzyme-linked immunospot assay. **Results:** The administration of HBsAg + cGAMP + SPD or HBsAg + K3-SPG + SPD significantly enhanced HBsAg-specific interferon-γ production by CD8 T cells from wild-type and HBV-Tg mice. The administration of HBsAg, cGAMP, and SPD increased serum HBsAb levels in wild-type and HBV-Tg mice. In HBV-Tg mice, the administration of SPD + cGAMP or SPD + K3-SPG with HBV vaccination significantly reduced HBsAg levels in the liver and serum.

**Conclusions:**

These results indicate that the combination of HBV vaccine adjuvant and SPD induces a stronger humoral and cellular immune response through T-cell activation. These treatments may support the development of a strategy to completely eliminate HBV.

## INTRODUCTION

Although an effective vaccine against HBV infection is currently being utilized, HBV remains a serious health problem worldwide. There are ~257 million people chronically infected worldwide and over 887,000 deaths every year according to a WHO report.^1^ Chronic hepatitis B (CHB) often causes cirrhosis and liver cancer.^2^ Treatments for CHB typically involve the life-long administration of approved drugs (eg, nucleotides) that are costly and require patient compliance. Thus, there is an urgent need for new treatments that can effectively control the HBV epidemic and eventually eradicate CHB.

HBV is a DNA virus that is converted into a covalently closed circular DNA in the host cell nucleus.^3^ Licensed antivirals target HBV reverse transcriptase activity but fail to eliminate covalently closed circular DNA. HBV covalently closed circular DNA elimination is currently achieved only by antiviral immune responses.^3^ However, HBV-specific T cells are scarce and functionally impaired in the context of chronic HBV infection most likely due to high amounts of circulating viral HBe antigen and HBsAg.^4^ A therapeutic vaccine combined with an adjuvant might restore a functional T-cell response and achieve anti-hepatitis B seroconversion while minimizing the risk of adverse effects. Therefore, a new approach using adjuvants is needed to break immune tolerance in CHB patients and enhance the functional HBV-specific immune response. Recently, we examined the adjuvant effect of cyclic guanosine monophosphate-AMP (cGAMP) and B/K CpG ODN (K3) wrapped with the nonagonistic Dectin-1 ligand, schizophyllan (K3-SPG) on HBV therapeutic vaccination.^5^,^6^ These adjuvants could induce HBV-specific humoral and cellular immune responses in wild-type (WT) and HBV-transgenic (HBV-Tg) mice through the enhancement of cytokine production and antigen-presenting function. However, the reduction in HBsAg levels in HBV-Tg mice was not sufficient after immunization.

Polyamines [including putrescine, spermidine (SPD), and spermine] are small, positively charged molecules that are involved in several processes in mammalian cells, such as gene transcription, mRNA translation, cell growth, and apoptosis.^7^,^8^ There is accumulating evidence that polyamines are novel autophagy inducers and longevity elixirs.^9^ Spermine increases autophagy by directly binding to the p53 and p21 promoters.^10^ Furthermore, spermine ameliorates ischemia/reperfusion injury in cardiomyocytes through the regulation of autophagy.^11^ Puleston et al^12^ found that boosting autophagy in older mice using SPD, which is also found naturally in many tissues, helped to restore the ability to create and maintain memory cells. In addition, mice treated with SPD acquired stronger immunity to influenza after vaccination than untreated mice of the same age. In the present study, we show that the administration of SPD induces stronger adjuvant-enhanced HBV-specific humoral and cellular immune responses in WT and HBV-Tg mice.

## METHODS

### Mice

WT male B10.D2 (H-2d) mice (age 8–12 wk; weight 25–30 g) were obtained from Japan SLC Inc. Mice of the HBsAg transgenic lineage 107-5D [official designation Tg (Alb-1, HBV) Bri66; inbred B10.D2, H-2d] in which the HBV envelope coding region is under control of the mouse albumin promoter were generously provided by Dr. Francis V. Chisari (Department of Molecular and Experimental Medicine, Scripps Research Institute, La Jolla, CA). All procedures were conducted in accordance with the National Institutes of Health Guide for the Care and Use of Laboratory Animals, and with the guidelines for the care and use of animals established by the Animal Care and Use Committee of Gifu University, Japan.

### Reagents

SPD ≥ 99% was obtained from Sigma-Aldrich. Distilled drinking water containing SPD (5 mM) was systemic administered daily. Yeast-derived recombinant HBsAg (r-HBsAg) vaccine containing the major surface protein of subtype adr (Bimmugen) containing aluminum was purchased from Meiji Seika Pharma. r-HBsAg which does not contain aluminum was obtained from Meridian Life Science Inc. Cyclic guanosine monophosphate–AMP (cGAMP) was purchased from InvivoGen. The K type of CpG-ODN (K3) (5′-ACTGACTCTCGAGCGTTCTC-3′) wrapped with the nanoparticulate CpG-ODN (K3-SPG) were generously provided by Ken J Ishii. The HBsAg peptide, IPQSLDSWWTSL, which binds to major histocompatibility complex class I molecules was synthesized at KURABO.

### Detection of specific antibodies against HBsAg

Serum samples were obtained from immunized mice. The concentration of HBsAb in serum was measured by an automated analyzer (HISCL-5000, Sysmex Corporation).

### Enzyme-linked immunospot (ELISpot) assay

The antigen-specific cellular immune response was assessed by ELISpot assay as described.^13^ Mice were subcutaneously inoculated on the tail base with HBsAg (3 μg/mouse), HBsAg (3 μg/mouse) + cGAMP (2 μg/mouse), or HBsAg (3 μg/mouse) + K3-SPG (10 μg/mouse) on days 0 and 10 or on days 0, 10, and 42. Single-cell suspensions were prepared from the whole spleen 17 or 56 days after the first immunization. A total of 3.0×10^5^ splenocytes/well were stimulated for 18 hours with 0–10 μg/mL of the HBsAg peptide IPQSLDSWWTSL in 96-well MultiScreen filter plates (Millipore) precoated with a monoclonal rat anti-interferon (IFN)-γ antibody (R4–6A2, BD Biosciences). The plates were washed and then incubated with a biotinylated polyclonal goat anti IFN-γ antibody (R&D Systems) and then with streptavidin-alkaline phosphatase. Spots were visualized by the addition of a 5-bromo-4-chloro-3-indolyl phosphatase solution (Sigma-Aldrich) and counted manually under a microscope (40× magnification). The number of cytokine-secreting cells was determined by a single blinded observer, and all data were generated by analyzing three separate wells per sample.

### Flow cytometric analysis of splenocytes

Splenocytes were isolated from the immunized mice as previously described.^14^ Cell viability and cell number were assessed using a trypan blue exclusion assay. For flow cytometry, 2×10^5^ splenocytes were stained with labeled antibodies using a standard protocol. The following antibodies were used: FITC-labeled CD4 monoclonal antibody (mAb) (clone GK1.5; BioLegend), APC-labeled anti-mouse CD8 mAb (clone 53–6.7; BioLegend), PE-labeled anti-mouse CD11a mAb (clone M17/4; BioLegend), PE-Cy7-labeled anti-mouse CD49d mAb (clone R1-2; BioLegend), FITC-labeled anti-mouse CD11b mAb (clone M1/70; BioLegend), PE-labeled anti-mouse CD11c mAb (clone N418; eBioscience), PE-Cy7-labeled anti-mouse CD86 mAb (clone GL-1; BioLegend), Pacific Blue–labeled anti-mouse CD40 mAb (clone 3/23; BioLegend), APC-labeled anti-mouse major histocompatibility complex II mAb (clone M5/114.15.2; BioLegend), PE-Cy7-labeled anti-mouse CD69 mAb (clone H1.2F3; eBioscience), PerCP/Cy5.5-labeled anti-mouse CD62L mAb (clone MEL-14; eBioscience), and Pacific Blue–labeled anti-mouse CD44 mAb (clone IM7; BioLegend). Data were acquired on a flow cytometer and data analysis was performed using FACSDiva software (BD Biosciences).

### Histological examination

Histopathological examination of the liver was performed 7 days after the second immunization or 14 days after the third immunization. The liver tissues were fixed in 10% formalin in PBS for 48 hours and embedded in paraffin. Tissue sections were deparaffinized, stained with hematoxylin and eosin and examined under light microscopy. The intrahepatic distribution of HBV antigens was assessed by the indirect immunoperoxidase method using an HBs antibody (Bioss Inc.).

### Immunocytochemistry

At the appropriate time points, the mice were killed by cervical dislocation and necropsy was performed. Liver tissues were collected, incubated in 10% buffered formalin and embedded in paraffin. Sections (4 µm thick) of the livers were stained with anti-CD4 antibody (1:500, catalog no. ab183685, Abcam) and anti-CD8 antibody (1:500, catalog no. ab217344, Abcam). These stains were using by BOND RX Fully Automated Research Stainer (Leica). The sections were evaluated using light microscopy. The scale bar for the high-power field represents 50 μm.

### Statistical analysis

Data are presented as the mean ± SEM. Differences between the experimental and control groups were analyzed using the Kruskal–Wallis test followed by the Scheffe *F* test. Statistical significance was indicated by *p* < 0.05.

## RESULTS

### SPD enhanced the cGAMP-induced cellular and humoral immune responses in HBsAg vaccination

**FIGURE 1:**
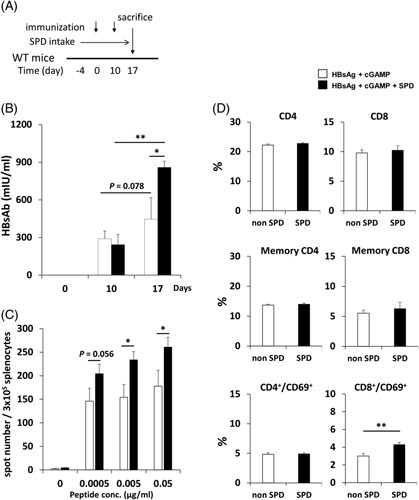
Cellular and humoral immune response in WT mice after immunization with r-HBsAg, cGAMP, and SPD. WT mice were immunized twice, on day 0 and day 10, with r-HBsAg (3 μg/mouse) + cGAMP (2 μg/mouse). SPD (5 mM) was administered in drinking water beginning 4 days before vaccination. Serum was obtained from immunized mice 10 and 17 days after immunization. Splenocytes were isolated from immunized mice 7 days after the second immunization. (A) Experimental schedule. (B) The concentration of HBsAb in serum from mice immunized twice. The results are shown as the mean ± SEM (3 mice/group) of 2 independent experiments. (C) Splenocytes from mice immunized twice were stimulated *ex vivo* with the IPQSLDSWWTSL peptide (0–0.05 μg/mL) and monitored for interferon-γ-secreting cells by ELISpot assay. The results are shown as the mean ± SEM (3 mice/group) of 2 independent experiments. (D) Splenocytes from immunized mice were stained for CD4, CD8, CD69, CD44, and CD62L. CD4^+^ and CD8^+^ T cells were gated for CD44 and CD62L analysis. * *p* < 0.05, ** *p* < 0.01. Abbreviations: cGAMP, cyclic guanosine monophosphate–AMP; SPD, spermidine; WT, wild-type.

The ability of SPD to induce HBsAb was examined after the second immunizations with r-HBsAg (3 μg/mouse) and cGAMP (2 μg/mouse) in WT mice (Figure 1A). Systemic administration of SPD significantly increased the HBsAb titer compared with the control (Figure 1B). Next, we assessed the ability of SPD to induce a cellular immune response to HBsAg. As shown in Figure 1C, the mice treated with SPD mounted strong cellular responses against HBsAg, as indicated by the significant expansion of IFN-γ-producing cells in response to *ex vivo* restimulation with HBsAg peptides. We also measured the proportions of CD4-positive or CD8-positive cells in the spleen. There were no differences between groups in the proportions of CD4-positive and CD8-positive cells and the proportion of memory CD4-positive and CD8-positive (CD44^high^/CD62L^low^) cells in the splenocyte population. However, the expression of CD69 by CD8 T cells was upregulated by the administration of SPD (Figure 1D). We also examined the ability of SPD to induce humoral and cellular immune responses upon vaccination with Beamgen (100 μL/mouse), which includes HBsAg and aluminum. As shown in Supplemental Figure 1B and C (http://links.lww.com/HC9/A220), SPD significantly enhanced humoral and cellular immune responses in Beamgen vaccination.

### SPD + cGAMP enhanced the HBsAg-specific immune response in HBV-Tg mice

**FIGURE 2:**
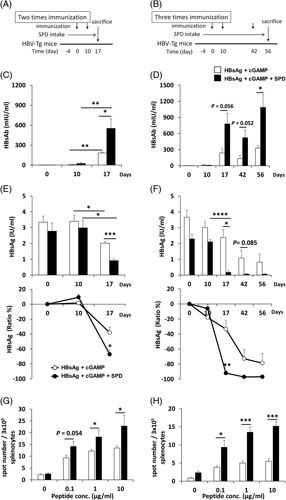
Cellular and humoral immune responses in HBV-Tg mice after immunization with r-HBsAg, cGAMP, and SPD. HBV-Tg mice were immunized 2 (A, C, E, and G) or 3 times (B, D, F, and H) with r-HBsAg + cGAMP or r-HBsAg + cGAMP+ SPD (3 mice/group). Serum was obtained from the mice 0, 10, 17, 42, and 56 days after the first immunization. (A and B) Experimental schedule. (C and D) HBsAb and (E and F) HBsAg levels in serum were measured after immunization. Splenocytes were isolated from HBV-Tg mice 7 days after the second immunization (G) or 14 days after the third immunization (H). Splenocytes were stimulated *ex vivo* with the HBsAg S28–39 peptide (0–10 μg/ml) and monitored for interferon-γ secreting cells by ELISpot assay. The results are shown as the mean ± SEM (3 mice/group). * *p* < 0.05, ** *p* < 0.01, *** *p* < 0.005, **** *p* < 0.001. Abbreviations: cGAMP, cyclic guanosine monophosphate–AMP; HBV-Tg, HBV-transgenic; SPD, spermidine.

Next, we evaluated the ability of SPD to induce HBsAg-specific cellular and humoral immune responses in HBV-Tg mice after immunization with r-HBsAg + cGAMP. HBV-Tg mice were treated 2 or 3 times with r-HBsAg + cGAMP or r-HBsAg + cGAMP + SPD (Figure 2A and B). Serum levels of HBsAb and HBsAg in HBV-Tg mice were measured at the time of vaccination, 7 days after the second immunization, and 14 days after the third immunization. As shown in Figure 2C, in HBV-Tg mice that received 2 doses of r-HBsAg + cGAMP, HBsAb levels were significantly increased by the administration of SPD. In addition, in HBV-Tg mice treated with 3 doses of r-HBsAg + cGAMP, HBsAb levels were increased by the administration of SPD (Figure 2D). HBsAg levels were significantly reduced in HBV-Tg mice treated with r-HBsAg + cGAMP + SPD compared with those treated with r-HBsAg + cGAMP (Figure 2E and F). To evaluate the HBsAg-specific cellular immune response, we performed ELISpot assays with samples from HBV-Tg mice immunized 2 or 3 times. Splenocytes from immunized HBV-Tg mice were stimulated with an HBsAg peptide (IPQSLDSWWTSL) at a higher concentration than that used to stimulate splenocytes from WT mice because HBV-Tg mice are tolerant to HBsAg. As shown in Figure 2G and H, HBV-Tg mice immunized with cGAMP + SPD mounted strong cellular responses against HBsAg, as indicated by the significant expansion of IFN-γ producing cells in response to *ex vivo* restimulation with HBsAg peptide.

### Phenotypes of CD4 and CD8 T cells after immunization with SPD

**FIGURE 3:**
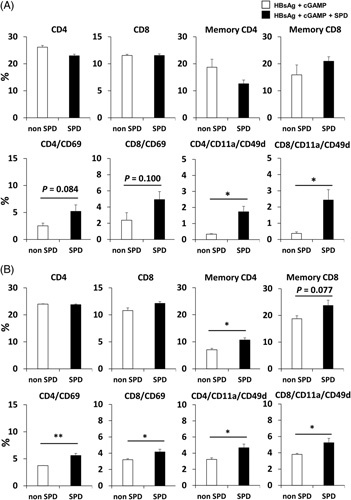
Phenotypes of lymphocytes in the spleen of HBV-transgenic mice after immunization. HBV-transgenic mice were immunized with r-HBsAg + cGAMP or r-HBsAg + cGAMP + SPD twice (A) or three times (B) (3 mice/group). Splenocytes were isolated from mice 7 days after the second immunization or 14 days after the third immunization. The percentages of CD4^+^, CD8^+^, memory (CD44^+^/CD62L^+^) T, CD69^+^ T, and CD11a^high^/CD49d^high^ T cells after immunization. * *p* < 0.05, ** *p* < 0.01. Abbreviations: cGAMP, cyclic guanosine monophosphate–AMP; SPD, spermidine.

Previous studies have reported that SPD activates the memory CD8^+^ T-cell response in aged mice in an autophagy-dependent manner.^12^ Therefore, in this study, to confirm that SPD activates the T-cell response, flow cytometry was performed using mouse splenocytes collected after the second or third immunization. HBV-Tg mice treated with SPD did not exhibit changes in the percentages of CD4^+^, CD8^+^, and memory (CD44^+^/CD62L^+^) T cells after 2 immunizations (Figure 3A and Supplemental Figure 2A, http://links.lww.com/HC9/A221). However, after 3 immunizations, a significant increase in memory CD4^+^ T cells was observed, and memory CD8^+^ T cells showed an increasing tendency (Figure 3B and Supplemental Figure 2B, http://links.lww.com/HC9/A221). Moreover, CD69 expression was significantly increased in CD4^+^ and CD8^+^ T cells after 3 immunizations (Figure 3B). Antigen-specific T cells are characterized by high expression of both CD11a and CD49d.^15^,^16^ Therefore, we stained splenocytes from immunized mice with anti-CD11a and anti-CD49d antibodies. The frequencies of CD11a^high^ and CD49d^high^ cells among CD4 and CD8 T cells were significantly increased in mice treated with SPD (Figure 3A and B).

### Immunization with HBsAg, cGAMP, and SPD-induced liver inflammation and decreased HBsAg levels in the liver

**FIGURE 4:**
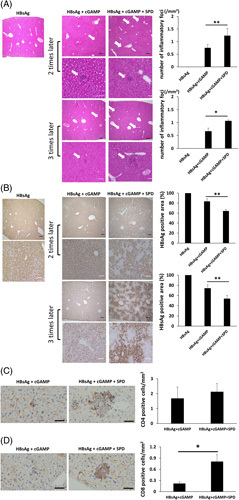
Histological examination of the liver in HBV-transgenic mice after immunization with r-HBsAg + cGAMP + SPD. (A) Representative micrographs of hematoxylin and eosin–stained liver sections 7 days after the second immunization or 14 days after the third immunization. Black scale bar: 300 µm. The white arrow indicates focal necrosis, and the image is enlarged as the lower square. The number of inflammatory foci is shown in the right panel. (B) The intrahepatic HBsAg content was determined by immunohistochemical staining for HBsAg (3 mice/group). Black scale bar: 300 µm, white scale bar: 100 µm. (C and D) Immunohistochemical staining for CD4-positive (C) or CD8-positive (D) cells in liver sections from experimental mice after treatments. The quantification of CD4-positive or CD8-positive cells was counted on all sections (scale bars, 50 μm; n=3 per group). * *p* < 0.05, ** *p* < 0.01. Abbreviations: cGAMP, cyclic guanosine monophosphate–AMP; SPD, spermidine.

Next, we performed a histological examination of liver tissues from HBV-Tg mice after immunization. Focal necrotic areas were observed in the liver of HBV-Tg mice immunized with r-HBsAg (3 μg/mouse) + cGAMP (2 μg/mouse) or r-HBsAg + cGAMP + SPD (Figure 4A). Moreover, the number of focal necrotic areas in mice immunized with r-HBsAg + cGAMP + SPD was significantly increased compared with that in mice immunized without SPD. HBsAg levels in the liver of mice immunized 3 times were significantly decreased by the administration of SPD, and the positively stained region was smaller than that after the second immunization (Figure 4B). Immunohistochemical analysis revealed that CD4-positive and CD8-positive cells mainly existed in the inflammatory foci of the liver (Figure 4C and D). SPD treatment significantly increased the number of CD8-positive cells in the liver after the immunization with HBsAg + cGAMP.

### SPD + K3-SPG enhanced the HBsAg-specific immune response in HBV-Tg mice

**FIGURE 5:**
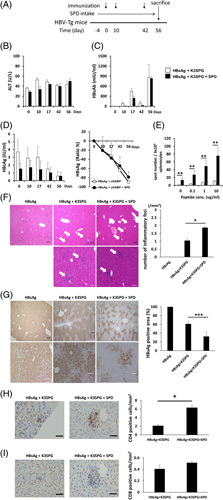
Effects of SPD on r-HBsAg and K3-SPG immunization in HBV-transgenic (HBV-Tg) mice. HBV-Tg mice were immunized with r-HBsAg (3 μg/mouse) + K3-SPG (10 μg/mouse) or r-HBsAg + K3-SPG + SPD (5 mM). Serum was obtained from the mice 0, 10, 17, 42, and 56 days after the first immunization. (A) Experimental schedule. (B) ALT, (C) HBsAb, and (D) HBsAg levels in serum were measured after immunization. (E) HBV-Tg mice were immunized 3 times (day 0, day 10, and day 42). Splenocytes were isolated from immunized mice 14 days after the third immunization. Splenocytes were stimulated *ex vivo* with the HBsAg S28–39 peptide (0–10 μg/mL) and monitored for interferon-γ secreting cells by ELISpot assay. The results are shown as the mean ± SEM (3 mice/group). (F) Representative micrographs of liver sections were collected 14 days after the third immunization and stained with hematoxylin and eosin. Black scale bar: 300 μm. The white arrow indicates focal necrosis, and the image is enlarged in the square photograph on the right side. (G) The intrahepatic HBsAg content was determined by immunohistochemical staining for HBsAg, black scale bar: 300 µm, white scale bar: 100 µm. (H and I) Immunohistochemical staining for CD4-positive (H) or CD8-positive (I) cells in liver sections from experimental mice after treatments. The quantification of CD4-positive or CD8-positive cells was counted on all sections (scale bars, 50 μm; n=3 per group).* *p* < 0.05, ** *p* < 0.01, *** *p* < 0.005. Abbreviations: cGAMP, cyclic guanosine monophosphate–AMP; HBV-Tg, HBV-transgenic; SPD, spermidine.

CpG-ODN has a powerful adjuvant effect in HBV vaccination. A recent report demonstrated that immunization with B/K CpG-ODN (K3) wrapped with the nonagonistic Dectin-1 ligand, schizophyllan (SPG), namely K3-SPG, is more effective at inducing the HBV-specific immune response than immunization with K3.^6^ We next examined whether the ability of K3-SPG to induce the HBV-specific immune response in HBV-Tg mice is enhanced by SPD. HBV-Tg mice were immunized with r-HBsAg (3 μg/mouse) + K3-SPG (10 μg/mouse) or r-HBsAg + K3-SPG + SPD 3 times to evaluate the ability of SPD to induce HBsAg-specific cellular and humoral immune responses (Figure 5A). SPD treatment did not affect ALT levels, HBsAb levels or HBsAg levels in the serum after immunization with r-HBsAg + K3-SPG (Figure 5B–D). In contrast, when the splenocytes of immunized mice were stimulated with the HBsAg peptide (IPQSLDSWWTSL), those from SPD-treated mice showed a significant increase in IFN-γ producing cells *ex vivo* (Figure 5E). Histological examination of liver tissue after immunization showed that the administration of SPD significantly increased the number of localized necrotic regions in K3-SPG-immunized mice (Figure 5F). As shown in Figure 5F, HBsAg expression in the liver of mice immunized with r-HBsAg + K3-SPG + SPD were significantly reduced compared with mice immunized with r-HBsAg + K3-SPG. Furthermore, the addition of SPD markedly reduced HBsAg expressions in the liver of K3-SPG-immunized mice (Figure 5G). As shown in Figure 5H and I, SPD treatment significantly increased the number of CD4-positive cells in the liver after the immunization with HBsAg + K3-SPG. However, the number of CD8-positive cells was not affected by SPD treatment in the immunization with HBsAg + K3-SPG.

## DISCUSSION

In the present study, we examined the effect of SPD on HBsAg vaccination in WT and HBV-Tg mice. The administration of SPD increased the adjuvant effect of cGAMP, K3-SPG, and aluminum on HBsAg vaccination. As a result, SPD treatment significantly enhanced the cellular and humoral immune responses of WT mice immunized with HBsAg + cGAMP, K3-SPG, or aluminum. HBV-Tg mice are immunologically tolerant to HBV antigens. However, vaccination with r-HBsAg + cGAMP + SPD or r-HBsAg + K3-SPG + SPD induced HBV-specific cellular and humoral immunity, even in HBV-Tg mice. In addition, these forms of immunization reduced HBsAg levels in the liver and serum of HBV-Tg mice.

In general, aluminum is commonly used for prophylactic vaccination against HBV. Recently, various studies have examined the adjuvant effect of TLR agonists in prophylactic and therapeutic vaccination for HBV.^17^,^18^ In particular, the development of therapeutic vaccines for chronic HBV infection has been actively pursued in many basic and clinical studies.^19^ In the current treatment for chronic hepatitis caused by HBV infection, oral nucleoside/nucleotide analogs are commonly used to suppress HBV replication. However, nucleotide analogs do not completely eliminate HBV from infected patients, who need to take nucleotide analogs continuously. Therefore, there is a need to develop a novel strategy to completely eliminate HBV from patients. In patients with chronic HBV infection, the HBV-specific immune response is quite weak.^20^ Breakthroughs in terms of immunological tolerance to HBV-specific antigen have led to the development of a novel strategy for the treatment of chronic HBV infection. In the present study, we demonstrated that the additional administration of SPD significantly enhanced the adjuvant effect of cGAMP, K3-SPG, and aluminum on HBsAg specific humoral and cellular immune responses in WT and HBV-Tg mice. The administration of HBsAg and SPD alone did not enhance HBsAg specific humoral and cellular immune responses (data not shown). There is accumulating evidence that polyamines are novel autophagy inducers and longevity elixirs.^9^ Boosting autophagy in older mice using SPD, which is also found naturally in many tissues, was shown to help restore the ability to create and maintain memory cells.^12^ In addition, mice treated with SPD acquired stronger immunity to influenza after vaccination than untreated mice of the same age. SPD administration increased the population of CD69-positive cells among CD4+ and CD8+ T cells (Figure 3). The increase in CD69-positive cells may lead to the enhancement of HBsAg-specific humoral and cellular immune responses.

HBV-Tg mice are a suitable animal model for the HBV carrier state because HBV-Tg mice harbor HBV from the neonatal period, and express HBV-related antigen in sera and liver throughout their lives.^21^ HBsAg-specific lymphocytes are not detected in these HBV-Tg mice *in situ*. Moreover, vaccination with HBsAg and complete Freund’s adjuvant does not induce the production of anti-HBsAb and HBsAg-specific lymphocytes in HBV-Tg mice.^21^ A previous study demonstrated that immunization with r-HBsAg + cGAMP or r-HBsAg + K3-SPG increased HBsAb levels and IFN-γ producing CD8 T cells, even in HBV-Tg mice.^5^,^6^ Both of cGAMP and K3-SPG induced HBsAg-specific immune responses through the enhancement of cytokine production and antigen presenting function. The treatment with cGAMP increased HBsAb level in the serum compared with the treatment with K3-SPG in HBsAg immunization. In the present study, 2 or 3 immunizations with r-HBsAg + cGAMP + SPD significantly increased serum HBsAb levels compared with immunization with r-HBsAg + cGAMP (Figure 2C). In addition, HBsAg-specific IFN-γ production in CD8 T cells of HBV-Tg mice was enhanced after the administration of r-HBsAg + cGAMP + SPD or r-HBsAg + K3-SPG + SPD (Figures 2G, H, 5E). Thus, SPD could increase the HBsAg-specific cellular immune response induced by vaccination with either r-HBsAg + cGAMP or r-HBsAg + K3-SPG. Moreover, histological examination revealed that the number of inflammatory foci in HBV-Tg mice immunized with r-HBsAg + cGAMP + SPD or r-HBsAg + K3-SPG + SPD was higher than that in mice immunized with r-HBsAg + cGAMP or r-HBsAg + K3-SPG (Figure 4A). In contrast, there was no inflammatory foci in the liver of WT mice immunized with r-HBsAg + cGAMP + SPD or r-HBsAg + K3-SPG + SPD (data not shown). Therefore, the increase in these foci may reflect the enhanced HBV-specific cellular immune response in HBV-Tg mice after immunization. Moreover, immunohistochemical analysis for the CD4 and CD 8 T cells revealed that CD4 and 8 T cells mainly existed in the inflammatory foci in the liver (Figures 4C, D, 5H, I). SPD treatment significantly increased the number of CD8 T cells in HBV-Tg mice immunized with r-HBsAg + cGAMP. In HBV-Tg mice immunized with r-HBsAg + cGAMP, the number of CD4 T cells increased by SPD treatment. Thus, SPD treatment may alter the phenotype of infiltrating cells into the liver in HBV-Tg mice immunized with different adjuvant. A recent report indicated that the primary goal of an HBV “functional cure” is sustained HBsAg loss.^22^ Reducing HBsAg expression may permit reconstitution of an immune response against HBV.^23^ In the present study, immunization with r-HBsAg + cGAMP + SPD significantly reduced HBsAg levels in the liver or serum compared with immunization with r-HBsAg + cGAMP (Figures 2 and 4). In addition, SPD markedly decreased HBsAg levels in the liver of HBV-Tg mice after immunization with r-HBsAg + K3-SPG. However, there was no difference in serum HBsAg levels of HBV-Tg mice between immunization with SPD and without SPD. This result may stem from the lack of change in HBsAb levels after additional SPD administration in HBV-Tg mice immunized with r-HBsAg + K3-SPG. Thus, additional SPD administration could reduce HBsAg levels in HBV-Tg mice and may lead to the development of a treatment for chronic HBV infection aimed at achieving a functional cure.

In conclusion, we evaluated the effect of SPD on HBV vaccination and demonstrated that vaccination with r-HBsAg + cGAMP + SPD enhances the induction of HBV-specific cellular and humoral immune responses in WT mice. These effects were also observed in HBV-Tg mice. In this study, SPD was shown to enhance the efficacy of multiple HBV vaccine adjuvants. The combination of SPD and HBV vaccine adjuvant may represent a strategy to completely eliminate HBV from patients.

## Supplementary Material
