# Supplementary figures and images for: Spermidine enhances the efficacy of adjuvant in HBV vaccination in mice
Source: Hepatol Commun. 2023 Mar 24;7(4):e0104. doi: 10.1097/HC9.0000000000000104 (PMC10043579; doi:10.1097/HC9.0000000000000104)

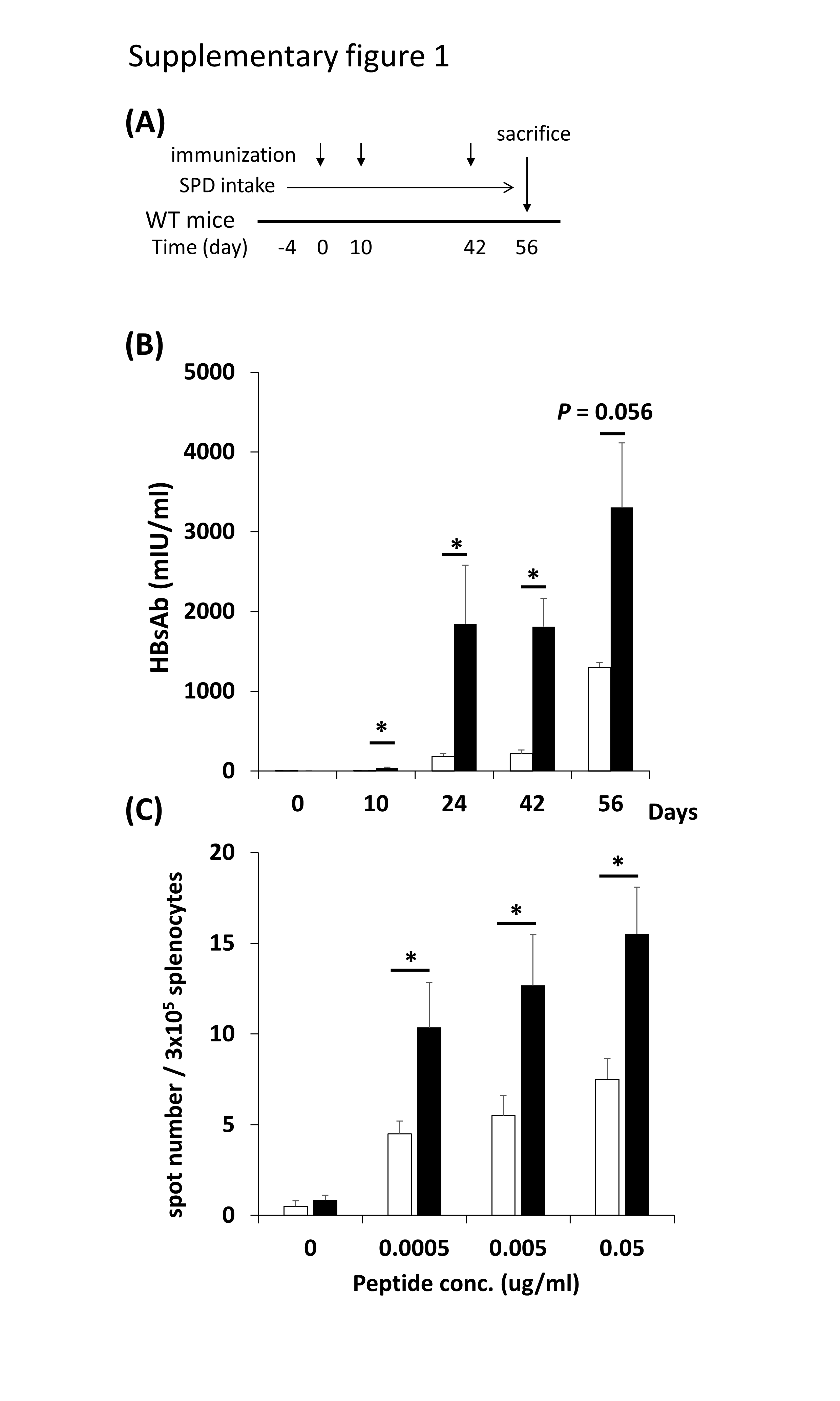

Supplement: Supplementary file 1 [file hc9-7-e0104-s001.tif]

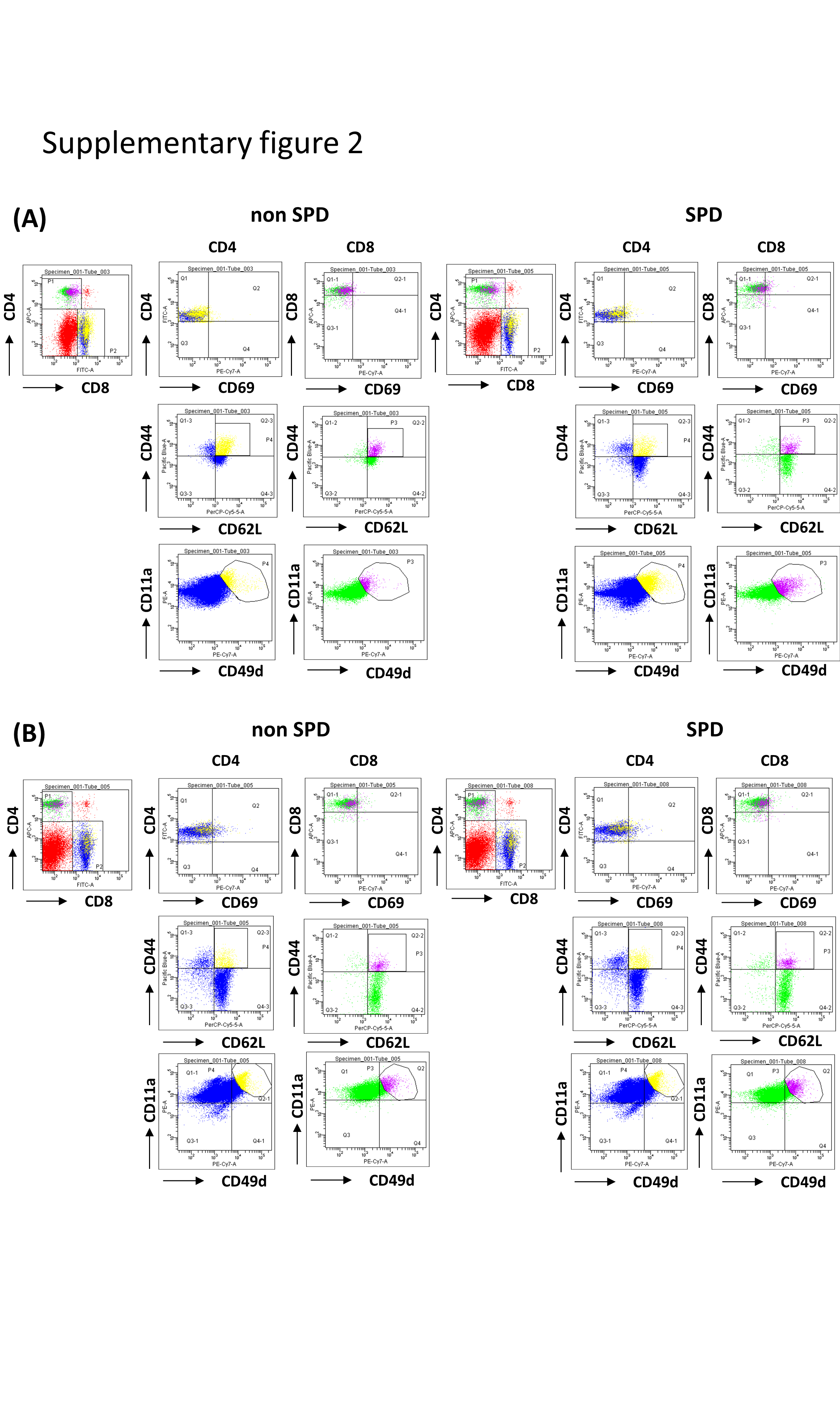

Supplement: Supplementary file 2 [file hc9-7-e0104-s002.tif]
